# Reliability of Universal-Platform-Based *Voice Screen* Application in AVQI Measurements Captured with Different Smartphones

**DOI:** 10.3390/jcm12124119

**Published:** 2023-06-18

**Authors:** Virgilijus Uloza, Nora Ulozaitė-Stanienė, Tadas Petrauskas, Kipras Pribuišis, Tomas Blažauskas, Robertas Damaševičius, Rytis Maskeliūnas

**Affiliations:** 1 Department of Otorhinolaryngology, Lithuanian University of Health Sciences, 50061 Kaunas, Lithuania; virgilijus.ulozas@lsmuni.lt (V.U.); tadas@petrauskas.co.uk (T.P.); kipras.pribuisis@lsmuni.lt (K.P.); 2 Faculty of Informatics, Kaunas University of Technology, 51368 Kaunas, Lithuania; tomas.blazauskas@ktu.lt (T.B.); robertas.damasevicius@ktu.lt (R.D.); rytis.maskeliunas@ktu.lt (R.M.)

**Keywords:** voice screen app, dysphonia screening, AVQI, smartphones

## Abstract

The aim of the study was to develop a universal-platform-based (UPB) application suitable for different smartphones for estimation of the Acoustic Voice Quality Index (AVQI) and evaluate its reliability in AVQI measurements and normal and pathological voice differentiation. Our study group consisted of 135 adult individuals, including 49 with normal voices and 86 patients with pathological voices. The developed UPB “*Voice Screen*” application installed on five iOS and Android smartphones was used for AVQI estimation. The AVQI measures calculated from voice recordings obtained from a reference studio microphone were compared with AVQI results obtained using smartphones. The diagnostic accuracy of differentiating normal and pathological voices was evaluated by applying receiver-operating characteristics. One-way ANOVA analysis did not detect statistically significant differences between mean AVQI scores revealed using a studio microphone and different smartphones (F = 0.759; *p* = 0.58). Almost perfect direct linear correlations (r = 0.991–0.987) were observed between the AVQI results obtained with a studio microphone and different smartphones. An acceptable level of precision of the AVQI in discriminating between normal and pathological voices was yielded, with areas under the curve (AUC) displaying 0.834–0.862. There were no statistically significant differences between the AUCs (*p* > 0.05) obtained from studio and smartphones’ microphones. The significant difference revealed between the AUCs was only 0.028. The UPB “*Voice Screen*” application represented an accurate and robust tool for voice quality measurements and normal vs. pathological voice screening purposes, demonstrating the potential to be used by patients and clinicians for voice assessment, employing both iOS and Android smartphones.

## 1. Introduction

Mobile communication devices such as smartphones or tablets are widely available to most of the global population, with the number of smartphone subscriptions expected to reach about 7145 billion by 2024 [1]. The increasing number of validated applications for smartphones in the field of general otorhinolaryngology and especially in a field related to voice assessment and management of voice disorders is permanently monitored in the literature [2,3,4,5,6]. Advances in smartphone technology and microphone quality offer an affordable and accessible alternative to studio microphones traditionally used for speech analysis, thus providing an effective tool for assessing, detecting, and caring for voice disorders [7,8,9].

The combination of variables in smartphone hardware and software may lead to differences between voice quality measures. Whether acoustic voice features recorded using smartphones sufficiently match the current gold standard for remote monitoring and clinical assessment with a studio microphone remains uncertain [7,10,11]. Some controversies on this matter in the literature still exist. Several studies found that smartphone-provided voice recordings and derived acoustic voice quality parameters are comparable to those derived using standard studio microphones [8,12,13,14]. Seung Jin Lee et al. found a significant correlation of several selected acoustic measures and no difference in the diagnostic ability between the Computerized Speech Lab and smartphone devices, although differences in several measures and higher cut-off scores of the smartphone were noted. Authors concluded that smartphones could be used as a screening tool for voice disorders [15]. On the other hand, using some other acoustic voice quality parameters could be discouraging [16]. Two recent studies found that none of the studied smartphones could replace the professional microphone in a voice recording to evaluate the six parameters analyzed, except for f_0_ and jitter. Moreover, passing a voice signal through a telecom channel induced both filter and noise effects which significantly impacted common acoustic voice quality measures [17,18].

Nowadays, multiparametric models for voice quality assessment are generally accepted to be more reliable and valid than single-parameter measures because they demonstrate stronger correlations with auditory–perceptual voice evaluation and are more representative of daily use patterns. For example, the Acoustic Voice Quality Index (AVQI) is a six-variable acoustic model for the multiparametric measurement, evaluating both the voiced parts of a continuous speech fragment and a sustained vowel (a:), developed by Maryn et al. in 2010 [19,20].

Multiple studies across different languages have attested to the reliability of the AVQI as a clinical voice quality evaluation tool. High consistency, concurrent validity, test–retest reliability, high sensitivity to voice quality changes through voice therapy, utility in discriminating across the perceptual levels of dysphonia severity, and adequate diagnostic accuracy with good discriminatory power of the AVQI in differentiating between normal and abnormal voice qualities were observed [20,21,22,23,24,25,26,27]. It is noteworthy that several studies have reported that sex and age do not affect the overall AVQI value, thus proving the perspectives for further generalization of this objective and quantitative voice quality measurement [27,28,29,30]. Therefore, nowadays, AVQI is considered a recognized-around-the-globe multiparametric construct of voice quality assessment for its clinical and research applications [31,32,33].

Several previous studies have proved the suitability of using smartphone voice recordings performed both in acoustically treated sound-proof rooms or in ordinary users’ environments to estimate the AVQI [4,9,11,14,27,34]. However, just a few studies in the literature provide data about AVQI realization using different applications for mobile communication devices [4,9,27]. The study by Grillo et al. in 2020 presented an application (*VoiceEvalU8*) that provided an automatic option for the reliable calculation of several acoustic voice measures and AVQIs on iOS and Android smartphones using the Praat source code and algorithms [4]. A user-friendly application/graphical user interface for the Kannada-speaking population was proposed by Shabnam et al. in 2022. The application provided a simplified output for AVQI cut-off values to depict the AVQI-based severity of dysphonia, which could be comprehendible by patients with voice disorders and health professionals [27]. The multilingual “*Voice Screen”* application developed by Uloza et al. allowed voice recording in clinical settings, automatically extracting acoustic voice features, estimating the AVQI result and displaying it alongside a recommendation to the user [9]. However, the “*Voice Screen”* application runs the iOS operating system, and that feature limits the usability only to iPhones, tablets, etc.

The results of the studies mentioned above enabled us to presume the feasibility of voice recordings captured with different smartphones for the estimation of AVQI. Consequently, the current research was designed to answer the following questions regarding the possibility of a smartphone-based “*Voice Screen*” application for AVQI estimation: (1) are the average AVQI values estimated by different smartphones consistent and comparable, and (2) are the diagnostic accuracy properties of different smartphone-estimated AVQIs relevant to differentiate normal and pathological voices? We hypothesize that using different smartphones for voice recordings and estimations of AVQI will be feasible for the quantitative voice assessment.

Therefore, the present study aimed to develop a universal-platform-based (UPB) application suitable for different smartphones for the estimation of AVQI and evaluate its reliability in AVQI measurements and normal/pathological voice differentiation.

## 2. Materials and Methods

All subjects gave their informed consent for inclusion before they participated in the study. The study was conducted in accordance with the Declaration of Helsinki of 1975, and the protocol was approved by the Kaunas Regional Ethics Committee for Biomedical Research (2022-04-20 No. BE-2-49).

The study group consisted of 135 adult individuals: 58 men and 77 women. The mean age of the study group was 42.9 (SD 15.26) years. They were all examined at the Department of Otolaryngology of the Lithuanian University of Health Sciences, Kaunas, Lithuania. The pathological voice subgroup consisted of 86 patients: 42 men and 44 women, with a mean age of 50.8 years (SD 14.3). They presented with a relatively common and clinically discriminative group of laryngeal diseases and related voice disturbances, i.e., benign and malignant mass lesions of the vocal folds and unilateral paralysis of the vocal fold. The normal voice subgroup consisted of 49 selected healthy volunteer individuals: 16 men and 33 women, mean age 31.69 (SD 9.89) years. This subgroup was collected following three criteria to define a vocally healthy subject: (1) all selected subjects considered their voice as normal and had no actual voice complaints and no history of chronic laryngeal diseases or voice disorders; (2) no pathological alterations in the larynx of the healthy subjects were found during video laryngoscopy; and (3) all these voice samples were evaluated as normal voices by otolaryngologists working in the field of voice. Demographic data of the study group and diagnoses of the pathological voice subgroup are presented in Table 1.

No correlations between the subject’s age, sex, and AVQI measurements were found in the previous study [28]. Therefore, in the present study, the control and patient groups were considered suitable for AVQI-related data analysis, despite these groups not being matched by sex and age.

### 2.1. Original Voice Recordings

Voice samples from each subject were recorded in a T-series sound-proof room for hearing testing (T-room, CATegner AB, Bromma, Sweden) using a studio oral cardioid AKG Perception 220 microphone (AKG Acoustics, Vienna, Austria). The microphone was placed at a 10.0 cm distance from the mouth, keeping a 90° microphone-to-mouth angle. Each participant was asked to complete two vocal tasks, which were digitally recorded. The tasks consisted of (1) sustaining phonation of the vowel sound (a:) for at least 4 s duration and (2) reading a phonetically balanced text segment in Lithuanian “Turėjo senelė žilą oželį” (“The granny had a small grey goat”). The participants completed both vocal tasks at a personally comfortable loudness and pitch. All voice recordings were captured with Audacity recording software (https://www.audacityteam.org/, accessed on 30 May 2023) at a sampling frequency of 44.1 kHz and exported in a 16-bit depth lossless “wav” audio file format onto the computer’s hard disk drive (HDD).

### 2.2. Auditory-Perceptual Evaluation

Five experienced physicians–laryngologists, who were all native Lithuanians, served as the rater panel. Blind to all relevant information regarding the subject (i.e., identity, age, gender, diagnosis, and disposition of the voice samples), they performed auditory–perceptual evaluations to quantify the vocal deviations, judging the voice samples into four ordinal severity classes of grade from the GRBAS scale (i.e., 0 = normal, 1 = slight, 2 = moderate, 3 = severe dysphonia) [35]. A detailed description of the auditory–perceptual evaluation is presented elsewhere [22].

### 2.3. Transmitting Studio Microphone Voice Recordings to Smartphones

The impact on voice recordings caused by technical differences in studio and smartphone microphones was averted by applying the filtration (equalization) of the already recorded flat frequency audio using the data from the smartphone frequency response curves. The filtered result would represent audio recorded with the selected smartphone. Using this method, the only variable affected was the frequency response, keeping other variables, i.e., room reflections, distance to the microphone, directionality, user loudness, and other variables, constant. Ableton DAW (digital audio workstation) was implemented as an editing environment, and the VST (virtual studio plugin) plugin MFreeformEqualizer by MeldaProduction (https://www.meldaproduction.com/MFreeformEqualizer/features, accessed on 4 June 2023) was used to import the frequency response datasets and equalize the frequencies according to the required frequency response. The MFreeformEqualizer filter quality was set to the extreme (highest available), with 0% curve smoothing. All the audio files were then re-exported as 44,100 Hz 16-bit wav files. With this method, the digital voice recordings obtained with a studio microphone were directly transmitted to different smartphones for analysis, avoiding not only the surrounding environment’s impact but also ideally synchronizing all voice samples throughout all devices without the need for additional audio synchronization methods to ensure that the exact same parts of vowels and speech were used for each smartphone’s analysis.

### 2.4. AVQI Estimation

For AVQI calculations, the signal processing of the voice samples was performed in the Praat software (version 5.3.57; https://www.fon.hum.uva.nl/praat/, accessed on 4 June 2023). Only voiced parts of the continuous speech were manually extracted and concatenated to the medial 3 s of the sustained (a) phonation. The voice samples were concatenated for auditory–perceptual judgment in the following order: text segment, a 2 s pause, followed by a 3 s sustained vowel /a/ segment. This chain of signals was used for acoustic analysis with the AVQI script version 02.02 developed for the program Praat https://www.vvl.be/documenten-en-paginas/praat-script-avqi-v0203?download=AcousticVoiceQualityIndexv.02.03.txt, accessed on 4 June 2023.

### 2.5. Development of a Universal-Platform-Based “Voice Screen” Application for Automated AVQI Estimation

The “*Voice Screen*” application for use with iOS operating devices was developed in the initial stage. Background noise monitoring, voice recording, and developed automated AVQI calculations were implemented in the application. Consequently, the “*Voice Screen*” application allowed voice recording, automatically extracted acoustic voice features, and displayed the AVQI result alongside a recommendation to the user [9].

**Figure 1:**
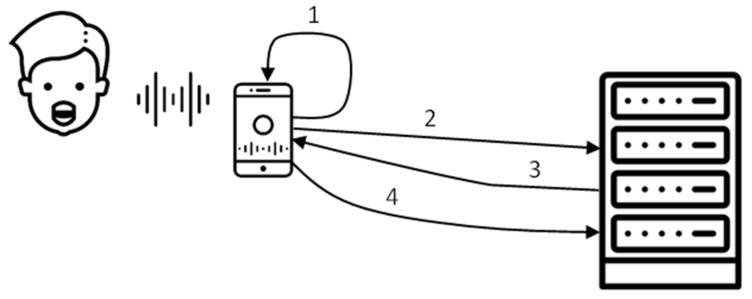
Structure of the system and flow of the operations.

The upgraded UPB version of the “*Voice Screen*” application, suitable for iOS and Android devices, was elaborated in the next stage. In this case, the calculation of the AVQI and its characteristics was performed on the server; therefore, the computationally costly sound processing was not dependent on the user’s device’s computational capabilities. We used the Flutter framework (https://flutter.dev/, accessed on 4 June 2023) to create our client application. It allowed for compiling applications for different platforms (devices and their operating systems) from a single code base. The framework ensured that the same algorithms ran on different devices and that no new software errors were introduced while porting the application. Currently, our application works with both iOS and Android devices. Figure 1 shows the structure of the system. The numbers in the picture depict the flow of the operations.

In the first step, the given smartphone (iOS or Android) records sound waves acquired while saying given phrases aloud. The sound waves are preprocessed (see Step 1 in Figure 1) in real-time. The preprocessing aims to clean the sound waves from pauses and ensure the minimum amount of sound suitable for further analysis. Step 2 sends the preprocessed sound wave to the server for further analysis. The server runs a Linux operating system and provides web services for software in Python. That software is based on the Praat (https://www.fon.hum.uva.nl/praat/, accessed on 4 June 2023) application ported into a Python library by the Parselmouth project (https://parselmouth.readthedocs.io/, accessed on 4 June 2023). We use this library to calculate AVQI and other sound characteristics used in AVQI calculation. In Step 3, the AVQI index and the related data are returned to the smartphone and displayed to the user. Step 4 is optional. If the user chooses to save the results, the sound waves and calculated characteristics are saved into the server’s database. No personal data relating to a specific person with the calculated AVQI and its parameters is saved on a server.

In the present study, the UPB “*Voice Screen*” application was installed on five different smartphones (namely, iPhone Pro Max 13, iPhone SE (iOS operating system), OnePlus 9 PRO, Samsung S22 Ultra, Huawei P50 pro (Android operating system)) used for AVQI estimation. The AVQI measures estimated with the “*Voice Screen*” application from voice recordings obtained from a flat frequency response studio microphone AKG Perception 220 were compared with AVQI results obtained using these smartphone devices.

### 2.6. Statistical Analysis

Statistical analysis was performed using IBM SPSS Statistics for Windows, version 20.0 (IBM Corp., Armonk, NY, USA) and MedCalc Version 20.118 (MedCalc Software Ltd., Ostend, Belgium). The chosen level of statistical significance was 0.05.

The data distribution was determined according to the normality law by applying the Shapiro–Wilk test of normality and calculating the coefficients of skewness and kurtosis. Student’s *t*-test was used to test the equality of means in normally distributed data [36]. An analysis of variance (ANOVA) was employed to determine if there were significant differences between the multiple means of the independent groups [37]. Cronbach’s alpha was used to measure the internal consistency of measures [38]. Pearson’s correlation coefficient was applied to assess the linear relationship between variables obtained from continuous scales. Spearman’s correlation coefficient was used to determine the relationship in ordinal results. Receiver operating characteristic (ROC) curves were used to obtain the optimal sensitivity and specificity at optimal AVQI cut-off points. The “area under the ROC curve” (AUC) served to calculate the possible discriminatory accuracy of AVQI performed with a studio microphone and different smartphones. A pairwise comparison of ROC curves, as described by De Long et al., was used to determine if there was a statistically significant difference between two or more variables when categorizing normal/pathological voices [39].

## 3. Results

### 3.1. Raters’ Perceptual Evaluation Outcomes

The rater panel demonstrated excellent inter-rater agreement (Cronbach’s α = 0.967) with a mean intra-class correlation coefficient of 0.967 between five raters (from 0.961 to 0.973).

### 3.2. AVQI Evaluation Outcomes

An individual smartphone AVQI evaluation displayed excellent agreement by achieving a Cronbach’s alpha of 0.984. The inter-smartphone AVQI measurements’ reliability was excellent, with an average Intra-class Correlation Coefficient (ICC) of 0.983 (ranging from 0.979 to 0.987).

The mean AVQI scores provided by different smartphones and a studio microphone can be observed in Table 2.

As shown in Table 2, the one-way ANOVA analysis did not detect statistically significant differences between mean AVQI scores revealed using different smartphones (F = 0.759; *p* = 0.58). Further Bonferroni analysis reaffirmed the lack of difference between the AVQI scores obtained from different smartphones (*p* = 1.0, estimated Bonferroni’s *p* for statistically significant difference *p* = 0.01). The mean AVQI differences ranged from 0.01 to 0.4 points when comparing different smartphones.

Almost perfect direct linear correlations were observed between the AVQI results obtained with a studio microphone and different smartphones. Pearson’s correlation coefficients ranged from 0.991 to 0.987 and can be observed in Table 3.

**Figure 2:**
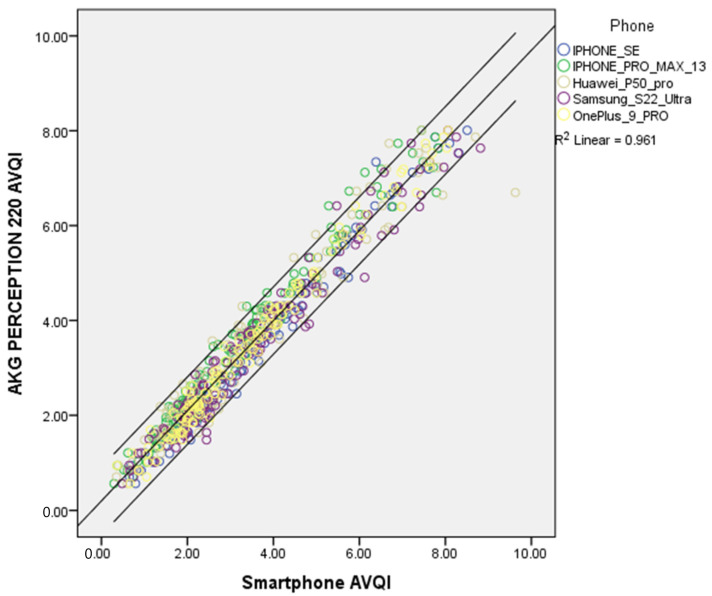
Scatterplot illustrating the correlation between the AVQI results obtained from the studio and different smartphones with a 95% confidence interval.

The relationships between the AVQI scores obtained with a studio microphone and different smartphones are graphically presented in Figure 2.

As demonstrated in Figure 2, it is evident that AVQI results obtained with different smartphones closely resemble the AVQI results obtained with a studio microphone, with very few data points outside of the 95% confidence interval (R^2^ = 0.961). Therefore, it is safe to conclude that the AVQI scores obtained with smartphones are directly compatible with the ones obtained with the reference studio microphone.

### 3.3. The Normal vs. Pathological Voice Diagnostic Accuracy of the AVQI Using Different Smartphones

**Figure 3:**
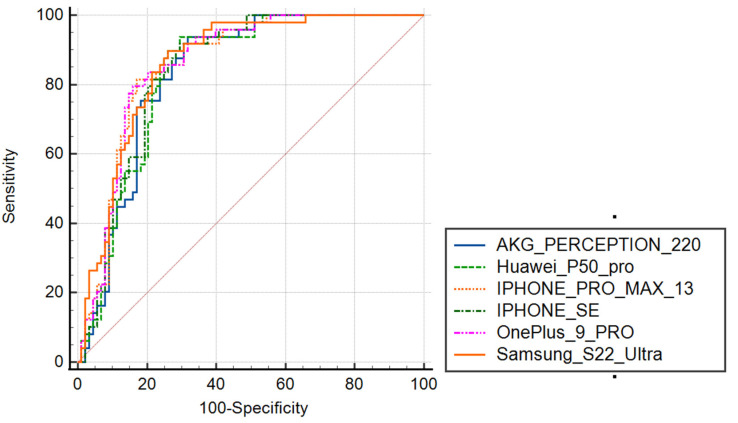
ROC curves illustrating the diagnostic accuracy of studio and different smartphone microphones in discriminating normal/pathological voices.

First, the ROC curves of AVQI obtained from a studio microphone and different smartphone voice recordings were inspected visually to identify optimum cut-off scores according to general interpretation guidelines [40]. All of the ROC curves were visually almost identical and occupied the largest part of the graph, clearly revealing their respectable power to discriminate between normal and pathological voices (Figure 3).

Second, as revealed by the AUC statistics analysis, a high level of precision of the AVQI in discriminating between normal and pathological voices was yielded with the suggested AUC = 0.800 threshold. The results of the ROC statistical analysis are presented in Table 4.

As demonstrated in Table 4, the ROC analysis determined the optimal AVQI cut-off values for distinguishing between normal and pathological voices for each smartphone. All employed microphones passed the proposed 0.8 AUC threshold and revealed an acceptable Youden-index value.

Third, a pairwise comparison of the significance of the differences between the AUCs revealed in the present study is presented in Table 5.

As shown in Table 5, a comparison of the AUCs-dependent ROC curves (AVQI measurements obtained from studio microphone and different smartphones), according to the test of DeLong et al., confirmed no statistically significant differences between the AUCs (*p* > 0.05). The most considerable observed difference between the AUCs was only 0.028. These results confirmed the compatible results of the AVQI’s diagnostic accuracy in differentiating normal vs. pathological voices when using voice recordings from a studio microphone and different smartphones.

## 4. Discussion

In the present study, the novel UPB “*Voice Screen*” application for the estimation of AVQI and detection of voice deteriorations in patients with various voice disorders and healthy controls was tested for the first time simultaneously with different smartphones. The AVQI was chosen for voice quality assessment because of some essential favorable features of this multiparametric measurement: the less vulnerability of the AVQI to environmental noise compared to other complex acoustic markers and the robustness of the AVQI regarding the interaction between acoustic voice quality measurements and room acoustics; there were no significant differences within subjects for both women and men when comparing the AVQI across different voice analysis programs [11,14,41]. Another essential attribute of the AVQI is that Praat is the only freely available program that estimates the AVQI. That eliminates the impact of possible software differences on AVQI computation.

In the present study, the results of the ANOVA analysis did not detect statistically significant differences between mean AVQI scores revealed using different smartphones (F = 0.759; *p* = 0.58). Moreover, the mean AVQI differences ranged from 0.01 to 0.4 points when comparing AVQI estimated with different smartphones, thus establishing a low level of variability. This corresponded with a value of 0.54 for the absolute retest difference of AVQI values proposed by Barsties and Maryn in 2013 [20,42]. Consequently, these outcomes of AVQI measurements with different smartphones were considered neither statistically nor clinically significant, justifying the possibility of practical use of the UPB “*Voice Screen*” app.

The correlation analysis showed that all AVQI measurements were highly correlated (Pearson’s r ranged from 0.991 to 0.987) across the devices used in the present study. This concurred with the literature data on the high correlation between acoustic voice features derived from studio microphones and smartphones and examined both for control participants and synthesized voice data [7,12,13,14].

Furthermore, analysis of the results revealed that the AVQI showed a remarkable ability to discriminate between normal and pathological voices as determined by auditory–perceptual judgment. The ROC analysis determined the optimal AVQI cut-off values for distinguishing between normal and pathological voices for each smartphone used. A remarkable precision of AVQI in discriminating between normal and pathological voices was yielded (AUC 0.834–0.862), resulting in an acceptable balance between sensitivity and specificity. These findings suggested that the AVQI was a reliable tool in differentiating normal/pathological voices independently of the voice recordings from tested studio microphones and different smartphones. The comparison of the AUC-dependent ROC curves (AVQI measurements obtained from studio microphone and different smartphones) demonstrated no statistically significant differences between the AUCs (*p* > 0.05), with the largest revealed difference between the AUCs of only 0.028. These results confirmed the compatible results of the AVQI diagnostic accuracy in differentiating normal vs. pathological voices when using voice recordings from studio microphone and different smartphones and presented remarkable importance from a practical point of view.

Several limitations of the present study have to be considered. Despite the encouraging results of the AVQI measurements, some individual discrepancies between AVQI results revealed with different smartphones still exist. Therefore, further research in a wide diversity of voice pathologies, including functional voice disorders, is needed to ensure the maximum comparability of acoustic voice features derived from voice recordings obtained with mobile communication devices and reference studio microphones. In the present study, the voice recordings were performed in a sound-proof room. However, in real clinical situations where environmental noise exists, the omni-directional built-in microphones of smartphones may induce different results. Therefore, further studies of the Voice Screen application’s performance with different smartphones in a real clinical setting are required to evaluate both the impact of the voice recording environment and the peculiarities of the microphones on the AVQI estimation in real clinical situations by performing simultaneous voice recordings with different smartphones. The outcomes of further studies will potentially make possible the results and improvements to be employed in healthcare applications.

Summarizing the results of the previous and present studies allows for the presumption that the performance of the novel UPB “*Voice Screen*” app using different smartphones represents an adequate and compatible performance of AVQI estimation. However, it is important to note that due to existing differences in recording conditions, microphones, hardware, and software, the results of acoustic voice quality measures may differ between recording systems [11]. Therefore, using the UPB “*Voice Screen*” app with some caution is advisable. For voice screening purposes, it is more reliable to perform AVQI measurements using the same device, especially when performing repeated measurements. Moreover, these bits of advice should be considered when comparing data of acoustic voice analysis between different voice recording systems, i.e., different smartphones or other mobile communication devices, and when using them for diagnostic purposes or monitoring voice treatment outcomes.

## 5. Conclusions

The UPB “*Voice Screen*” app represents an accurate and robust tool for voice quality measurement and normal vs. pathological voice screening purposes, demonstrating the potential to be used by patients and clinicians for voice assessments, employing both iOS and Android smartphones.

## Figures and Tables

**Table 1:** Demographic data of the study group.

Diagnosis	*n*	Age	
		Mean	SD
Normal voice	49	31.69	9.89
Mass lesions of vocal folds	49	44.39	12.4
Vocal fold cancer	11	65.09	7.71
Chronic hyperplastic laryngitis	10	55.9	7.34
Unilateral vocal fold paralysis	6	40.83	12.77
Bilateral vocal folds paralysis	4	52.75	12.61
Functional dysphonia	2	39	24.04
Reflux laryngitis	2	57	15.56
Parkinson’s disease	2	71.5	9.19
Total	135	42.92	15.26

Abbreviation: SD—standard deviation.

**Table 2:** Comparison of the mean AVQI results obtained with different smartphones and studio microphone.

Microphone	*n*	Mean AVQI	Std. Deviation	F	*p*
AKG Perception 220	135	3.43	1.83	0.759	0.58
iPhone SE		3.56	1.86		
iPhone Pro Max 13		3.16	1.83		
Huawei P50 pro		3.37	1.96		
Samsung S22 Ultra		3.52	1.93		
OnePlus 9 PRO		3.42	1.86		

Abbreviation: AVQI—acoustic voice quality index.

**Table 3:** Correlations of AVQI scores obtained with studio microphone and different smartphones.

Microphones		iPhone SE	iPhone Pro Max 13	Huawei P50 pro	Samsung S22 Ultra	OnePlus 9 PRO
AKG Perception 220	r	0.991	0.987	0.970	0.979	0.992
	*p*	0.001	0.001	0.001	0.001	0.001
	n	135	135	135	135	135

Abbreviations: r—Pearsons’s correlation coefficient; *p*—statistical significance.

**Table 4:** Statistics illustrating the accuracy the AVQI differentiating normal and pathological voices recorded using studio and different smartphones’ microphones.

AVQI	AUC	Cut-Off	Sensitivity %	Specificity %	Youden-Index J
AKG Perception 220	0.834	3.27	93.88	68.18	0.62
iPhone SE	0.844	3.23	91.84	70.45	0.62
iPhone Pro Max 13	0.858	2.14	81.63	82.95	0.65
Huawei P50 pro	0.835	3.08	93.88	70.45	0.64
Samsung S22 Ultra	0.862	2.93	89.8	73.86	0.64
OnePlus 9 PRO	0.86	2.3	79.59	84.09	0.64

Abbreviations: AVQI acoustic voice quality index, AUC area under the curve.

**Table 5:** A pairwise comparison of the significance of differences between the AUCs.

p	AKG Perception 220	iPhone SE	iPhone Pro Max 13	Huawei P50 Pro	Samsung S22 Ultra	OnePlus 9 Pro
AKG Perception 220	-	0.163	0.099	0.966	0.11	0.086
iPhone SE	0.163	-	0.367	0.579	0.282	0.863
iPhone Pro Max 13	0.099	0.367	-	0.268	0.718	0.863
Huawei P50 pro	0.966	0.579	0.268	-	0.223	0.256
Samsung S22 Ultra	0.11	0.282	0.718	0.223	-	0.863
OnePlus 9 PRO	0.086	0.863	0.863	0.256	0.863	-

Abbreviations: AUC area under the curve.

## Data Availability

The data presented in this study are available on request from the corresponding author.

## References

[1] Mobile Network Subscriptions Worldwide. 2028. https://www.statista.com/statistics/330695/number-of-smartphone-users-worldwide/.

[2] Casale M., Costantino A., Rinaldi V., Forte A., Grimaldi M., Sabatino L., Oliveto G., Aloise F., Pontari D., Salvinelli F. (2018). Mobile applications in otolaryngology for patients: An update. Laryngoscope Investig. Otolaryngol..

[3] Eleonora M.C.T., Lonigro A., Gelardi M., Kim B., Cassano M. (2021). Mobile Applications in Otolaryngology: A Systematic Review of the Literature, Apple App Store and the Google Play Store. Ann. Otol. Rhinol. Laryngol..

[4] Grillo E.U., Wolfberg J. (2020). An Assessment of Different Praat Versions for Acoustic Measures Analyzed Automatically by VoiceEvalU8 and Manually by Two Raters. J. Voice.

[5] Boogers L.S., Chen B.S.J., Coerts M.J., Rinkel R.N.P.M., Hannema S.E. Mobile Phone Applications Voice Tools and Voice Pitch Analyzer Validated with LingWAVES to Measure Voice Frequency. *J. Voice*
**2022**. https://www.sciencedirect.com/science/article/pii/S0892199722003186.

[6] Kojima T., Hasebe K., Fujimura S., Okanoue Y., Kagoshima H., Taguchi A., Yamamoto H., Shoji K., Hori R. (2021). A New iPhone Application for Voice Quality Assessment Based on the GRBAS Scale. Laryngoscope.

[7] Fahed V.S., Doheny E.P., Busse M., Hoblyn J., Lowery M.M. Comparison of Acoustic Voice Features Derived from Mobile Devices and Studio Microphone Recordings. *J. Voice*
**2022**. https://www.sciencedirect.com/science/article/pii/S0892199722003125.

[8] Awan S.N., Shaikh M.A., Awan J.A., Abdalla I., Lim K.O., Misono S. Smartphone Recordings are Comparable to “Gold Standard” Recordings for Acoustic Measurements of Voice. *J. Voice*
**2023**. https://www.sciencedirect.com/science/article/pii/S0892199723000310.

[9] Uloza V., Ulozaite-Staniene N., Petrauskas T. (2023). An iOS-based VoiceScreen application: Feasibility for use in clinical settings-a pilot study. Eur. Arch. Otorhinolaryngol..

[10] Munnings A.J. (2020). The Current State and Future Possibilities of Mobile Phone “Voice Analyser” Applications, in Relation to Otorhinolaryngology. J. Voice.

[11] Maryn Y., Ysenbaert F., Zarowski A., Vanspauwen R. (2017). Mobile Communication Devices, Ambient Noise, and Acoustic Voice Measures. J. Voice.

[12] Kardous C.A., Shaw P.B. (2014). Evaluation of smartphone sound measurement applications. J. Acoust. Soc. Am..

[13] Manfredi C., Lebacq J., Cantarella G., Schoentgen J., Orlandi S., Bandini A., DeJonckere P.H. (2017). Smartphones Offer New Opportunities in Clinical Voice Research. J. Voice.

[14] Grillo E.U., Brosious J.N., Sorrell S.L., Anand S. (2016). Influence of Smartphones and Software on Acoustic Voice Measures. Int. J. Telerehabil..

[15] Lee S.J., Lee K.Y., Choi H., Lee S.J., Lee K.Y., Choi H. (2018). Clinical Usefulness of Voice Recordings using a Smartphone as a Screening Tool for Voice Disorders. Commun. Sci. Disord..

[16] Schaeffler F., Jannetts S., Beck J.M. Reliability of clinical voice parameters captured with smartphones—Measurements of added noise and spectral tilt. Proceedings of the 20th Annual Conference of the International Speech Communication Association INTERSPEECH 2019.

[17] Marsano-Cornejo M., Roco-Videla Á. (2022). Comparison of the Acoustic Parameters Obtained with Different Smartphones and a Professional Microphone. Acta Otorrinolaringol. ESP.

[18] Pommée T., Morsomme D. Voice Quality in Telephone Interviews: A preliminary Acoustic Investigation. *J. Voice*
**2022**. https://www.sciencedirect.com/science/article/pii/S0892199722002685.

[19] Maryn Y., De Bodt M., Roy N. (2010). The Acoustic Voice Quality Index: Toward improved treatment outcomes assessment in voice disorders. J. Commun. Disord..

[20] Barsties B., Maryn Y. (2012). The Acoustic Voice Quality Index. Toward expanded measurement of dysphonia severity in German subjects. HNO.

[21] Hosokawa K., Barsties B., Iwahashi T., Iwahashi M., Kato C., Iwaki S., Sasai H., Miyauchi A., Matsushiro N., Inohara H. (2017). Validation of the Acoustic Voice Quality Index in the Japanese Language. J. Voice.

[22] Uloza V., Petrauskas T., Padervinskis E., Ulozaitė N., Barsties B., Maryn Y. (2017). Validation of the Acoustic Voice Quality Index in the Lithuanian Language. J. Voice.

[23] Kankare E., Barsties V., Latoszek B., Maryn Y., Asikainen M., Rorarius E., Vilpas S., Ilomäki I., Tyrmi J., Rantala L. (2020). The acoustic voice quality index version 02.02 in the Finnish-speaking population. Logop. Phoniatr. Vocol..

[24] Englert M., Lopes L., Vieira V., Behlau M. (2022). Accuracy of Acoustic Voice Quality Index and Its Isolated Acoustic Measures to Discriminate the Severity of Voice Disorders. J. Voice.

[25] Yeşilli-Puzella G., Tadıhan-Özkan E., Maryn Y. (2022). Validation and Test-Retest Reliability of Acoustic Voice Quality Index Version 02.06 in the Turkish Language. J. Voice.

[26] Englert M., Latoszek B.B.V., Behlau M. Exploring the Validity of Acoustic Measurements and Other Voice Assessments. *J. Voice*
**2022**. https://www.sciencedirect.com/science/article/pii/S0892199721004392.

[27] Shabnam S., Pushpavathi M., Gopi Sankar R., Sridharan K.V., Vasanthalakshmi M.S. A Comprehensive Application for Grading Severity of Voice Based on Acoustic Voice Quality Index v.02.03. *J. Voice*
**2022**. https://www.sciencedirect.com/science/article/pii/S0892199722002454.

[28] Latoszek B.B.V., Ulozaitė-Stanienė N., Maryn Y., Petrauskas T., Uloza V. (2019). The Influence of Gender and Age on the Acoustic Voice Quality Index and Dysphonia Severity Index: A Normative Study. J. Voice.

[29] Batthyany C., Maryn Y., Trauwaen I., Caelenberghe E., van Dinther J., Zarowski A., Wuyts F. (2019). A case of specificity: How does the acoustic voice quality index perform in normophonic subjects?. Appl. Sci..

[30] Jayakumar T., Benoy J.J., Yasin H.M. (2022). Effect of Age and Gender on Acoustic Voice Quality Index Across Lifespan: A Cross-sectional Study in Indian Population. J. Voice.

[31] Jayakumar T., Benoy J.J. Acoustic Voice Quality Index (AVQI) in the Measurement of Voice Quality: A Systematic Review and Meta-Analysis. *J. Voice*
**2022**. https://www.sciencedirect.com/science/article/pii/S0892199722000844.

[32] Batthyany C., Latoszek B.B.V., Maryn Y. Meta-Analysis on the Validity of the Acoustic Voice Quality Index. *J. Voice*
**2022**.

[33] Saeedi S., Aghajanzade M., Khatoonabadi A.R. (2022). A Literature Review of Voice Indices Available for Voice Assessment. JRSR.

[34] Uloza V., Ulozaitė-Stanienė N., Petrauskas T., Kregždytė R. (2021). Accuracy of Acoustic Voice Quality Index Captured with a Smartphone—Measurements with Added Ambient Noise. J. Voice.

[35] Dejonckere P.H., Bradley P., Clemente P., Cornut G., Crevier-Buchman L., Friedrich G., Van De Heyning P., Remacle M., Woisard V. (2001). A basic protocol for functional assessment of voice pathology, especially for investigating the efficacy of (phonosurgical) treatments and evaluating new assessment techniques. Guideline elaborated by the Committee on Phoniatrics of the European Laryngological Society (ELS). Eur. Arch. Otorhinolaryngol..

[36] Senn S., Richardson W. (1994). The first *t*-test. Stat. Med..

[37] McHugh M.L. (2011). Multiple comparison analysis testing in ANOVA. Biochem. Med..

[38] Cho E. (2016). Making Reliability Reliable: A Systematic Approach to Reliability Coefficients. Organ. Res. Methods.

[39] Hanley J.A., McNeil B.J. (1982). The meaning and use of the area under a receiver operating characteristic (ROC) curve. Radiology.

[40] Dollaghan C.A. (2007). The Handbook for Evidence-Based Practice in Communication Disorders.

[41] Bottalico P., Codino J., Cantor-Cutiva L.C., Marks K., Nudelman C.J., Skeffington J., Shrivastav R., Jackson-Menaldi M.C., Hunter E.J., Rubin A.D. (2020). Reproducibility of Voice Parameters: The Effect of Room Acoustics and Microphones. J. Voice.

[42] Lehnert B., Herold J., Blaurock M., Busch C. (2022). Reliability of the Acoustic Voice Quality Index AVQI and the Acoustic Breathiness Index (ABI) when wearing COVID-19 protective masks. Eur. Arch. Otorhinolaryngol..

